# Rhinoconjunctivitis among Adolescents in Kuwait and Associated Risk Factors: A Cross-Sectional Study

**DOI:** 10.1155/2019/3981064

**Published:** 2019-11-11

**Authors:** Ali H. Ziyab, Yaser M. Ali

**Affiliations:** ^1^Department of Community Medicine and Behavioral Sciences, Faculty of Medicine, Kuwait University, Kuwait City, Kuwait; ^2^Department of Internal Medicine, Mubarak Al-Kabeer Hospital, Ministry of Health, Kuwait City, Kuwait

## Abstract

Rhinoconjunctivitis is a public health problem that causes major illness and disability worldwide. Epidemiological studies intended to determine the burden of rhinoconjunctivitis in Kuwait are limited. Hence, this study sought to estimate the prevalence of rhinoconjunctivitis among adolescents in Kuwait and explore its association with different risk factors. Schoolchildren aged 11–14 years (*n* = 3,864) were enrolled in a cross-sectional study. Parents completed questionnaires regarding their children's clinical history and symptoms of rhinoconjunctivitis and relevant exposures. Associations were assessed using Poisson regression with robust variance estimation, and adjusted prevalence ratios (aPRs) and 95% confidence intervals (CIs) were estimated. The 12-month (current) prevalence estimates of rhinitis, rhinoconjunctivitis, and severe rhinoconjunctivitis were 28.6% (1,040/3,643), 13.5% (497/3,689), and 1.2% (44/3,689), respectively. The prevalence of current rhinoconjunctivitis symptoms was higher in boys compared to girls (aPR = 1.19, 95% CI: 1.01–1.41). Parental history of rhinitis and asthma showed positive associations with rhinoconjunctivitis in offspring. Trend analyses showed that rhinoconjunctivitis prevalence decreased with increasing numbers of total siblings (aPR = 0.92, *P*_trend_ < 0.001) and older siblings (aPR = 0.90, *P*_trend_ < 0.001). Rhinoconjunctivitis is common among adolescents in Kuwait and its epidemiology is similar to that found in western countries.

## 1. Introduction

Rhinitis is a common allergic disease that is characterized by adverse nasal symptoms, such as sneezing, itching, rhinorrhea, and/or nasal congestion [1]. These nasal symptoms often occur in conjunction with itchy, red, and watery eyes (conjunctivitis) [1, 2]. Although rhinoconjunctivitis, the co-occurrence of allergic nasal and ocular symptoms, is not considered a life-threatening disease, it is a major source of morbidity among children and adults. It has been shown that rhinoconjunctivitis is a risk factor for poor asthma control, negatively affects social life, and has detrimental effects on academic performance among children and work productivity among adults [3]. In addition to its impact on the quality of life and wellbeing, rhinoconjunctivitis has major economic ramifications for individuals and society [4].

Results of the international study of asthma and allergies in childhood (ISAAC) indicated that rhinoconjunctivitis variably affects children across the globe, with prevalence estimates ranging from 1.0% in India to 45.1% in Paraguay among adolescents aged 13/14 years [5]. In Kuwait, the prevalence of current rhinoconjunctivitis symptoms among adolescents was estimated to be 10.7%, which is slightly lower than the ISAAC averaged global prevalence estimate of 14.6% in those aged 13/14 years [5]. Moreover, increasing trends in rhinoconjunctivitis symptoms have been documented by the ISAAC and other investigations [6, 7]. Although literature on rhinoconjunctivitis continues to grow, many nations still lack essential epidemiological data regarding the disease burden. Given the limited empirical data on the epidemiology of rhinoconjunctivitis in Kuwait, this study sought to provide a recent estimate of rhinoconjunctivitis prevalence among adolescents in Kuwait and assess its association with different risk factors.

## 2. Methods

### 2.1. Study Setting, Design, and Participants

Kuwait is a small country in the Middle East, bordering the Persian (Arabian) Gulf, with land area of around 18,000 km^2^. A school-based, cross-sectional study was conducted by enrolling schoolchildren (*n* = 3,864) attending public middle schools from all six school districts in Kuwait [8]. The study sample mainly included students between 11 and 14 years old. Recruitment of schoolchildren into the study was done during the 2016/2017 school year (September 2016 to May 2017) and the first semester of the 2017/2018 school year (September to December 2017). A representative sample of schoolchildren was selected using a stratified two-stage cluster sampling method. In brief, given that public schools in Kuwait follow a single-sex system, a random sample of schools was selected from two sex-stratified sampling frames that included all public middle schools in Kuwait. Detailed sampling methodology and study setting is described by Ziyab [8]. Ethical approval for the current study was obtained from the Standing Committee for Coordination of Health and Medical Research, Ministry of Health, Kuwait (no. 2016/451). The study was conducted in accordance with principles and guidelines of the Declaration of Helsinki for medical research involving human subjects.

A study-specific questionnaire and a standardized questionnaire (i.e., the ISAAC questionnaire [9]) were sent home with the children for parental/guardian completion and were returned after completion to the school authorities. Information on the demographic data, lifestyle factors, environmental exposures, and clinical history and symptoms of allergic diseases of both the children and their parents were collected by the questionnaires. Written informed consent for study participation was obtained from each child's parents or legal guardians.

### 2.2. Definitions

The following core questions from the ISAAC questionnaire were used to define study outcomes:

1. In the past 12 months, has your child had a problem with sneezing or a runny or blocked nose, when he or she DID NOT have a cold or the flu?
2. In the past 12 months, has this nose problem been accompanied by itchy-watery eyes?
3. In the past 12 months, how much did this nose problem interfere with your child's daily activities? (Possible answers are the following: not at all, a little, a moderate amount, a lot.)
4. Has your child ever been diagnosed by a doctor with rhinitis?

We used criteria developed by the ISAAC to define the study outcomes [5]. Question 1 was used to estimate the current (12-month) prevalence of rhinitis symptoms. Affirmative responses to both questions 1 and 2 were used to ascertain the presence of current rhinoconjunctivitis symptoms. Positive responses to questions 1 and 2 and the answer “a lot” to question 3 were used to assess the prevalence of current severe rhinoconjunctivitis symptoms. Moreover, an affirmative response to question 4 was used to estimate the lifetime prevalence of parent-reported doctor-diagnosed rhinitis. To further understand the seasonal variations in the prevalence of rhinoconjunctivitis symptoms, affected participants reported the month(s) during which they experienced rhinoconjunctivitis symptoms in the past 12 months. Furthermore, in children with current rhinoconjunctivitis symptoms, perennial rhinoconjunctivitis was defined as the occurrence of symptoms for more than 9 months in the past 12 months [10].

### 2.3. Ascertainment of Exposure and Covariate Variables

The questionnaires completed by the parent/guardians gathered information on relevant exposures and covariates. Body mass index (BMI), a measure of general adiposity, markedly changes in children with growth; hence, we estimated the BMI-for-age z-scores (standard deviation (SD) scores) using the World Health Organization (WHO) growth reference for those aged between 5 and 19 years [11]. Using cutoff values developed by the WHO, the BMI-for-age score was categorized as follows: underweight (thinness): <−2 SD, normal: −2 to 1 SD, overweight: >1 to 2 SD, and obese: >2 SD [11]. Exposure to environmental tobacco smoke (ETS) was assessed by inquiring whether any member of the household smokes cigarettes or tobacco-related products inside the home. Two separate questions were asked to ascertain exposure to household cats and dogs during infancy: “Did you have a cat/dog in your home during the first year of this child's life?” Moreover, two separate questions were asked to determine current (past 12 months) exposure to household cats and dogs: “In the past 12 months, have you had a cat/dog in your home?” Breastfeeding status, categorized as ever versus never, was determined by asking whether the child was ever directly fed at the breast during infancy. The following question stem was used to determine maternal/paternal history of rhinitis, asthma, and eczema: “Has the child's mother/father ever been diagnosed with rhinitis/asthma/eczema by a doctor?” Information regarding the child's total number of siblings and number of older and younger siblings was collected by the questionnaire.

### 2.4. Statistical Analysis

All statistical analyses were conducted using SAS 9.4 (SAS Institute, Cary, North Carolina, USA). The statistical significance level was set to *α* = 0.05 for all association analyses. Frequencies and proportions of the categorical variables and the medians and 5th and 95th percentiles of the quantitative variables were determined. To assess whether the analytical study sample (*n* = 3,689, i.e., participants with complete information regarding rhinoconjunctivitis status) was representative of the total study sample (*n* = 3,864), we compared proportions of categorical variables (using *χ*^2^ tests) and means of continuous variables (using *t*-tests) across these two samples. The current (12-month) prevalence of rhinoconjunctivitis symptoms, current prevalence of rhinitis symptoms, current prevalence of severe rhinoconjunctivitis symptoms, and lifetime prevalence of parent-reported doctor-diagnosed rhinitis were estimated, along with their binomial 95% confidence intervals (CIs). Moreover, among affected individuals, monthly prevalence of rhinoconjunctivitis symptoms for the past 12 months was reported according to the four northern hemisphere meteorological seasons of the year (Winter: December–February; Spring: March–May; Summer: June–August; Fall: September–November).

The crude and adjusted associations were assessed by applying a modified Poisson regression with robust variance estimation using the GENMOD procedure in SAS 9.4 to estimate and infer the prevalence ratios (PRs) and their 95% CIs [12]. Variables that demonstrated possible association with current rhinoconjunctivitis symptoms (main outcome variable) in the crude models (i.e., *p* value≤ 0.2, as suggested by Maldonado and Greenland [13]) were simultaneously entered into the multivariable regression models. Regardless of statistical significance, sex and age were included as potential confounders in all multivariable regression models. Two analytical approaches were applied to assess the associations between the numbers of total, older, and younger siblings and current rhinoconjunctivitis symptoms: the variables were treated as (i) categorical (0, 1, 2, 3, ≥4 siblings, with 0 siblings group being the reference) and (ii) quantitative to infer the trends per additional sibling. While assessing the association between the number of older siblings and rhinoconjunctivitis symptoms, the number of younger siblings was included as a covariate in the regression model, and vice versa. Moreover, the association between perennial rhinoconjunctivitis symptoms and severe rhinoconjunctivitis symptoms was evaluated.

## 3. Results

### 3.1. Description of Study Population

The current study enrolled a total of 3,864 schoolchildren (2,169 girls and 1695 boys). Of these, 3,689 (95.5%) had information regarding rhinoconjunctivitis status. The analytical study sample and the total study sample were similar in all characteristics investigated (Table 1). The median (5th, 95th percentile) age of the study participants was 12 (11, 14) years. 6.2% and 13.1% of the study participants were exposed to household cats in infancy and in the past 12 months, respectively, whereas only 2.2% and 3.1% of children were exposed to household dogs in infancy and in the past 12 months, respectively (Table 1).

### 3.2. Prevalence of Rhinoconjunctivitis and Seasonal Variations

The lifetime prevalence estimate of parent-reported doctor-diagnosed rhinitis was 25.1% (917/3,652), with more boys being affected than girls (28.4% versus 22.5%, *p* value <0.001; Table 2). The current (12-month) prevalence estimates of rhinitis, rhinoconjunctivitis, and severe rhinoconjunctivitis were 28.6% (1,040/3,643), 13.5% (497/3,689), and 1.2% (44/3,689), respectively. Prevalence estimates of current rhinitis (31.6% versus 26.1%, *p* value <0.001) and current rhinoconjunctivitis (15.3% versus 12.0%, *p* value 0.004) were higher among boys compared to girls, whereas there was not difference between boys and girls in regard to the prevalence of severe rhinoconjunctivitis (1.4% versus 1.0%, *p* value = 0.274; Table 2).

**Figure 1:**
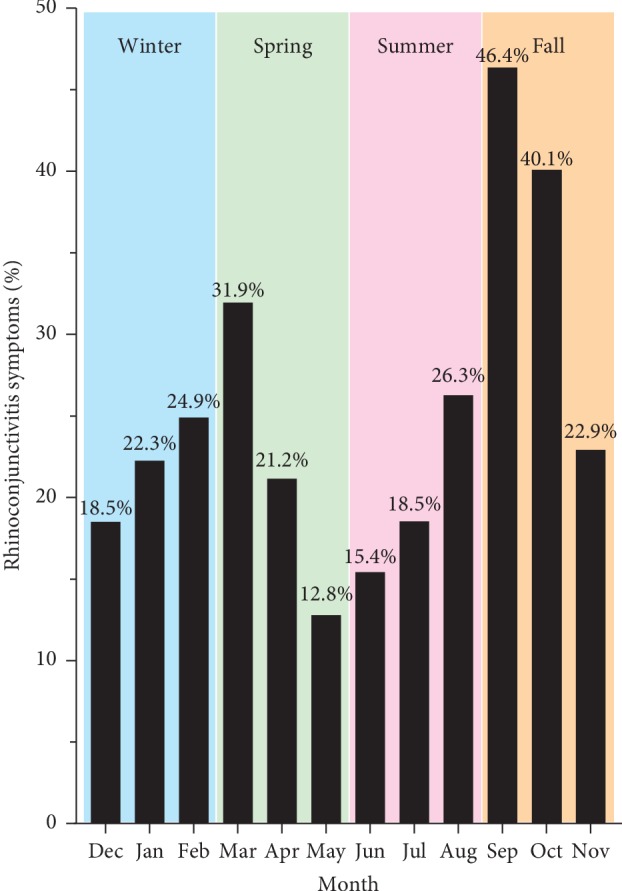
Monthly and seasonal variations in the prevalence of rhinoconjunctivitis symptoms. The shown prevalence estimates were calculated among individuals reporting rhinoconjunctivitis symptoms in the past 12 months.

Figure 1 shows the monthly and seasonal variations in rhinoconjunctivitis symptoms. Two peaks in symptoms were observed, specifically in spring (March-April) and fall (September-October), with prevalence of rhinoconjunctivitis symptoms being highest during early fall. Lowest prevalence estimates of symptoms were observed in the months of May and June.

### 3.3. Factors Associated with Rhinoconjunctivitis

Associations between personal attributes and risk factors and current rhinoconjunctivitis symptoms are presented in Table 3. After adjusting for potential confounders, the prevalence of current rhinoconjunctivitis symptoms was higher in boys compared to girls (adjusted PR (aPR) = 1.19, 95% CI: 1.01–1.41). In the crude analysis, exposure to household dog during infancy was associated with increased prevalence of current rhinoconjunctivitis symptoms (PR = 1.65, 95% CI: 1.09–2.51); however, after adjustment for the effects of potential confounders, this association lost statistical significance (aPR = 1.39, 95% CI: 0.92–2.09; Table 3). Parental history of rhinitis (aPR = 3.14, 95% CI: 2.53–3.90) and parental history of asthma (aPR = 1.26, 95% CI: 1.05–1.50) showed strong associations with current rhinoconjunctivitis symptoms in offspring, whereas parental history of eczema did not demonstrate association with current rhinoconjunctivitis symptoms in offspring (aPR = 1.09, 95% CI: 0.91–1.31; Table 3).

The results of the association analyses of the numbers of total, older, and younger siblings and current rhinoconjunctivitis symptoms are presented in Table 4. The trend analyses (per additional sibling) showed that the prevalence of current rhinoconjunctivitis symptoms decreased with increasing numbers of total siblings (aPR = 0.92, 95% CI: 0.88–0.96, *P*_trend_ < 0.001) and older siblings (aPR = 0.90, 95% CI: 0.85–0.94, *P*_trend_ < 0.001). Association between number of younger siblings and current rhinoconjunctivitis symptoms did not gain statistical significance (aPR = 0.94, 95% CI: 0.89–1.00, *P*_trend_=0.050; Table 4). Compared to having no (zero) older siblings, having four or more (≥4) older siblings was associated with a reduced prevalence of having current rhinoconjunctivitis symptoms (aPR = 0.59, 95% CI: 0.44–0.80).

**Figure 2:**
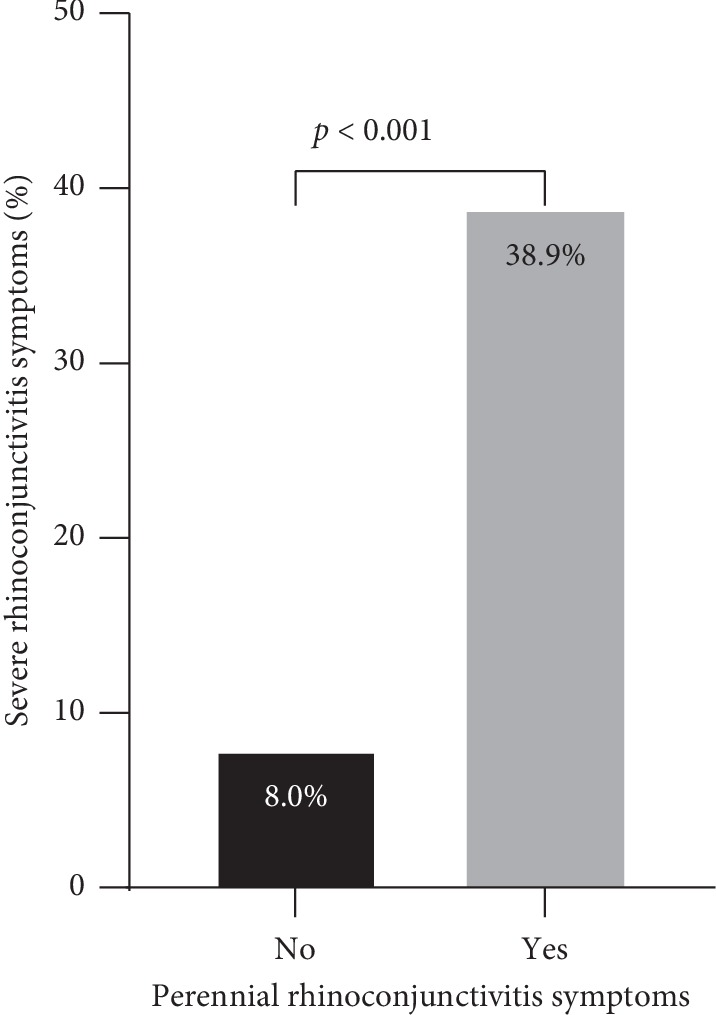
Prevalence of severe rhinoconjunctivitis symptoms according to perennial rhinoconjunctivitis symptoms. Participants were considered to have perennial rhinoconjunctivitis if symptoms were reported for more than 9 months in the past 12 months; otherwise, participants were not considered to have perennial rhinoconjunctivitis symptoms.

A further analysis showed that having perennial rhinoconjunctivitis symptoms was associated with reporting severe rhinoconjunctivitis symptoms (Figure 2). In particular, the prevalence of severe rhinoconjunctivitis symptoms was higher in those classified as having perennial rhinoconjunctivitis symptoms compared to those without perennial rhinoconjunctivitis symptoms (38.9% versus 8.0%, *p* value <0.001; Figure 2).

## 4. Discussion

This large cross-sectional study estimated the prevalence of rhinoconjunctivitis among adolescents in Kuwait and assessed its association with various risk factors. The prevalence of current rhinoconjunctivitis symptoms was 13.5%, with more boys being affected than girls (15.3% versus 12.0%). Moreover, the current severe rhinoconjunctivitis symptoms were reported by 1.2% of the study participants, which was equally reported by boys (1.4%) and girls (1.0%). Seasonal variations in rhinoconjunctivitis symptoms were observed, with peaks in spring (March-April) and fall (September-October). Risk factors that demonstrated possible associations with increased rhinoconjunctivitis symptoms included having household dog during infancy and parental history of rhinitis and asthma. In contrast, decreasing trends in the prevalence of rhinoconjunctivitis symptoms were observed in relation to increasing numbers of total and older siblings. Moreover, having perennial rhinoconjunctivitis symptoms was associated with increased prevalence of severe rhinoconjunctivitis symptoms.

Very limited studies are available on the epidemiology of rhinoconjunctivitis in Kuwait. The estimated prevalence of current rhinoconjunctivitis symptoms in this study (13.5%) is similar to the global average estimate (14.6%) in 2002 from the ISAAC study among adolescents aged 13/14 years [5]. Moreover, the estimate of current rhinoconjunctivitis (13.5%) in this study is slightly higher than a previous estimate among adolescents in Kuwait (10.7%) [5] and lower than an estimate among young adults in Kuwait (20.4%) [14]. This study estimated the prevalence of current severe rhinoconjunctivitis symptoms to be 1.2%, which is similar to the global average of 1.0% [5]. The estimated prevalence of current rhinitis symptoms (28.6%) is similar to a previous estimate from Kuwait (27.6%) [15] and close to the global average of 31.7% [5].

Seasonal variations in the prevalence of rhinoconjunctivitis symptoms were observed in the current study, with peaks occurring in spring (March-April) and fall (September-October). These observations are in agreement with a previous study from Kuwait, which showed that rhinitis-related hospital referrals peaked during two periods in Kuwait (i.e., April-May and September-October), with the fall peak being the highest [16]. The aforementioned study concluded that the September-October peak in rhinitis is related to a peak in total pollen, specifically the pollination of Chenopodiaceae species. Moreover, the observed seasonal variations in the current study are in agreement with prior studies from countries in the northern hemisphere [17–19].

In our study, there was a male predominance in regard to rhinoconjunctivitis symptoms. Such an observation contradicts the common observation in which a male predominance of rhinoconjunctivitis prevalence in childhood switches to a female predominance in adolescence [20, 21]. A large meta-analysis showed that rhinitis is more common in females compared to males during adolescence (pooled male-to-female ratio: 0.91, 95% CI: 0.86–0.95) [22]. However, when the results of the meta-analysis were stratified by region, a male predominance in rhinitis prevalence persisted beyond childhood for Asian studies only [22]. Moreover, a previous study from Kuwait conducted among adolescents aged 13/14 years showed that rhinitis symptoms were more common in males compared to females (31.1% versus 24.1%, *p* value <0.001) [15]. Hence, these results are in agreement with our observation of higher prevalence of rhinoconjunctivitis symptoms among males compared to females. Possible mechanisms explaining this sex disparity include differences in sex hormones. Estrogen may stimulate proinflammatory cytokines and increase the susceptibility to atopy in females, whereas androgens may act as immune suppressor hormones against allergic inflammation in males [20, 23, 24]. Nevertheless, the exact mechanisms underlying the observed sex-switchover during puberty in rhinoconjunctivitis and other allergic diseases remain unclear. Moreover, factors leading to higher prevalence estimates among adolescent males compared to females in Asian studies, including the current study, are under-investigated and warrant further corroboration.

In regard to potential risk factors, exposure to household dogs during infancy showed a trend for a positive association with current rhinoconjunctivitis symptoms. This association has been reported previously [25–27], and plausibly is explained by the fact that pet-keeping, including cats and dogs, is linked to increased household pet allergen and endotoxin and microbial exposures that might modulate the risk of allergic diseases [28, 29]. Moreover, in the current study, parental history of doctor-diagnosed rhinitis and asthma showed strong positive associations with current rhinoconjunctivitis symptoms. Such parental effects have been widely reported and are explained by the influence of genetics and epigenetics as well as the shared environment [30–32].

Furthermore, we showed that increased numbers of total and older siblings, and to a lesser extent number of younger siblings, are associated with reduced prevalence of rhinoconjunctivitis symptoms. Such observations of inverse associations are in agreement with the scientific literature [33–35]. The “hygiene hypothesis,” formulated by Strachan in 1989 to explain the protective effects of having a higher number of siblings on allergic diseases [33], postulates that the recent reduced infection rate and endotoxin exposure and increased household and personal cleanliness are related to an increased risk of allergic diseases [36]. Hence, the “sibling effects” phenomenon suggests that having a greater number of siblings, specifically older siblings, is a protective factor against allergic disease, which was initially explained by the unhygienic contact with older siblings leading to immune maturation through frequent infections in early life [35, 37]. However, a competing hypothesis suggests that in utero programming could explain the observed effects of siblings [35, 38]. In general, the effects of older siblings could reflect prenatal and/or postnatal programming, whereas associations with younger siblings are likely explained by postnatal factors.

A major strength of the current study is the representative and large study sample, which allowed for the estimation of rhinoconjunctivitis prevalence among schoolchildren throughout Kuwait. Moreover, using questions from the ISAAC standardized questionnaire [30] and applying disease definitions similar to those used in the ISAAC [5] increased the comparability of our results to international findings. Misclassification of disease is a recognized limitation of large population-based epidemiological studies that usually lead to overestimation of symptoms. However, given that the prevalence of current rhinoconjunctivitis symptoms in this study (13.5%) is in agreement with global results (ISAAC: 14.6% [5]) and close to a previous estimate from Kuwait (10.7% [15]), misclassification of the outcome is not of a major concern in the current study. Selection bias could also be a concern in large population-based cross-sectional studies; however, the possibility of selection bias affecting the results of our study is low because the response proportion was high (i.e., 73.9%, 3,864/5,228). Moreover, it is essential to indicate that our analysis aimed to assess associations between different exposures and rhinoconjunctivitis rather than to infer causal relationships.

## 5. Conclusions

The current study showed that rhinoconjunctivitis is common among adolescents in Kuwait and its burden resembles that of western nations. The higher prevalence of rhinoconjunctivitis among adolescent boys compared to girls observed in our study disagrees with studies from western countries; however, it is in line with Asian studies. Such an observation needs further corroboration. The observed positive associations of exposure to household dogs and parental history of asthma and rhinitis with rhinoconjunctivitis and the inverse association between the number of siblings and rhinoconjunctivitis are in agreement with the current state of knowledge. Overall, our study provided extensive epidemiological data on the burden of rhinoconjunctivitis among adolescents in Kuwait.

## Figures and Tables

**Table 1:** Characteristics of the total study sample and the analytical study sample.

Variables	Total study sample (*n* = 3864)	Analytical study sample (*n* = 3689)^§^
*Sex, n (%)*		
Female	2169 (56.1)	2061 (55.9)
Male	1695 (43.9)	1628 (44.1)

*Age (years)*		
Median (5^th^, 95^th^ percentile)	12.0 (11.0, 14.0)	12.0 (11.0, 14.0)

*BMI-for-age groups, n (%)*		
Underweight (<−2 SD)	219 (5.8)	211 (5.8)
Normal (−2 to 1 SD)	1517 (40.1)	1450 (40.1)
Overweight (>1 to 2 SD)	961 (25.3)	916 (25.3)
Obese (>2 SD)	1089 (28.8)	1039 (28.7)
Missing, *n*	78	73

*Mode of birth, n (%)*		
Vaginal	3106 (81.8)	2979 (81.6)
Cesarean section	692 (18.2)	670 (18.4)
Missing, *n*	66	40

*Breastfeeding ever, n (%)*		
Yes	2894 (76.3)	2777 (76.3)
Missing, *n*	72	49

*Cat exposure in infancy, n (%)*		
Yes	232 (6.1)	227 (6.2)
Missing, *n*	35	18

*Current cat exposure, n (%)*		
Yes	500 (13.1)	479 (13.1)
Missing, *n*	36	20

*Dog exposure in infancy, n (%)*		
Yes	85 (2.2)	82 (2.2)
Missing, *n*	32	17

*Current dog exposure, n (%)*		
Yes	119 (3.1)	113 (3.1)
Missing, *n*	28	14

*ETS exposure, n (%)*		
Yes	1755 (45.8)	1682 (45.7)
Missing, *n*	28	12

*Parental history of rhinitis* ^*∗*^ *, n (%)*		
Yes	1736 (46.4)	1700 (46.8)
Missing, *n*	126	59

*Parental history of asthma* ^†^ *, n (%)*		
Yes	1238 (32.9)	1207 (33.1)
Missing, *n*	102	39

*Parental history of eczema* ^‡^ *, n (%)*		
Yes	904 (24.1)	880 (24.2)
Missing, *n*	108	46

BMI: body mass index; SD: standard deviation; ETS: Environmental tobacco smoke. ^§^Sample of participants with complete information regarding rhinoconjunctivitis (i.e., excluding 175 subjects with incomplete information regarding rhinoconjunctivitis). ^*∗*^Maternal and/or paternal history of doctor-diagnosed rhinitis. ^†^Maternal and/or paternal history of doctor-diagnosed asthma. ^‡^Maternal and/or paternal history of doctor-diagnosed eczema.

**Table 2:** Prevalence of ever doctor-diagnosed rhinitis and current symptoms of rhinitis, rhinoconjunctivitis, and severe rhinoconjunctivitis in the total analytical sample and stratified by sex.

	% (*n*/total)	95% CI	Sex difference *p*-value^*∗*^
*Ever doctor-diagnosed rhinitis*			
Total	25.1 (917/3652)	23.7–26.5	
Males	28.4 (459/1614)	26.2–30.6	
Females	22.5 (458/2038)	20.6–24.3	<0.001

*Current rhinitis*			
Total	28.6 (1040/3643)	27.1–30.0	
Males	31.6 (509/1611)	29.3–33.9	
Females	26.1 (531/2032)	24.2–28.0	<0.001

*Current rhinoconjunctivitis*			
Total	13.5 (497/3689)	12.4–14.6	
Males	15.3 (249/1628)	13.6–17.0	
Females	12.0 (248/2061)	10.6–13.4	0.004

*Current severe rhinoconjunctivitis*			
Total	1.2 (44/3689)	0.8–1.5	
Males	1.4 (23/1628)	0.8–2.0	
Females	1.0 (21/2061)	0.6–15	0.274

CI: confidence interval. ^*∗*^Comparing prevalence in males and females using chi-squared tests.

**Table 3:** Crude and adjusted associations between personal attributes and risk factors and current rhinoconjunctivitis.

	Rhinoconjunctivitis, % (*n*/total)	Crude PR (95% CI)	Adjusted PR^§^ (95% CI)
*Sex*			
Female	12.0 (248/2061)	1.00 (ref.)	1.00 (ref.)
Male	15.3 (249/1628)	1.27 (1.08–1.50)	1.19 (1.01–1.41)

*Age (years)*			
Median (5^th^, 95^th^ percentile)	—	1.00 (0.94–1.07)	1.01 (0.94–1.08)
*BMI-for-age groups*			
Underweight (<−2 SD)	10.4 (22/211)	0.82 (0.54–1.26)	0.83 (0.55–1.26)
Normal (−2 to 1 SD)	12.6 (183/1450)	1.00 (ref.)	1.00 (ref.)
Overweight (>1 to 2 SD)	13.3 (122/916)	1.06 (0.85–1.31)	1.08 (0.87–1.33)
Obese (>2 SD)	15.8 (164/1039)	1.25 (1.03–1.52)	1.17 (0.96–1.42)

*Mode of birth*			
Vaginal	13.0 (387/2979)	1.00 (ref.)	1.00 (ref.)
Cesarean section	15.7 (105/670)	1.21 (0.99–1.47)	1.09 (0.89–1.33)

*Breastfeeding ever*			
No	13.9 (120/863)	1.00 (ref.)	—
Yes	13.4 (374/2777)	0.96 (0.79–1.16)	—

*Cat exposure in infancy*			
No	13.4 (463/3444)	1.00 (ref.)	—
Yes	14.5 (33/227)	1.08 (0.78–1.50)	—

*Current cat exposure*			
No	13.1 (419/3190)	1.00 (ref.)	1.00 (ref.)
Yes	15.9 (76/479)	1.21 (0.97–1.51)	1.17 (0.93–1.48)

*Dog exposure in infancy*			
No	13.3 (477/3590)	1.00 (ref.)	1.00 (ref.)
Yes	22.0 (18/82)	1.65 (1.09–2.51)	1.39 (0.92–2.09)

*Current dog exposure*			
No	13.4 (477/3562)	1.00 (ref.)	—
Yes	15.0 (17/113)	1.12 (0.72–1.76)	—

*ETS exposure*			
No	12.5 (249/1995)	1.00 (ref.)	1.00 (ref.)
Yes	14.6 (246/1682)	1.17 (0.99–1.38)	1.13 (0.95–1.33)

*Parental history of rhinitis* ^*∗*^			
No	6.3 (121/1930)	1.00 (ref.)	1.00 (ref.)
Yes	21.7 (368/1700)	3.45 (2.84–4.20)	3.14 (2.53–3.90)

*Parental history of asthma* ^†^			
No	10.6 (258/2443)	1.00 (ref.)	1.00 (ref.)
Yes	19.2 (232/1207)	1.82 (1.55–2.14)	1.26 (1.05–1.50)

*Parental history of eczema* ^‡^			
No	11.9 (328/2763)	1.00 (ref.)	1.00 (ref.)
Yes	18.2 (160/880)	1.53 (1.29–1.82)	1.09 (0.91–1.31)

ETS: Environmental tobacco smoke; PR: prevalence ratio; CI: confidence interval; BMI: body mass index; SD: standard deviation; ref.: reference. ^§^Variables that had a *p* value ≤0.2 in the crude model were simultaneously included in the adjusted (multivariable) model, except for age and sex, which were included in all adjusted model. In addition to the shown variables, “total number of siblings” was included in the multivariable model as it had a *p* value ≤0.2 in the crude model. ^*∗*^Maternal and/or paternal history of doctor-diagnosed rhinitis. ^†^Maternal and/or paternal history of doctor-diagnosed asthma. ^‡^Maternal and/or paternal history of doctor-diagnosed eczema.

**Table 4:** Crude and adjusted associations between the total number of siblings, number of older siblings, and number of younger siblings and current rhinoconjunctivitis.

	Rhinoconjunctivitis, % (*n*/total)	Crude PR^*∗*^ (95% CI)	Adjusted PR^†^ (95% CI)
*Total siblings*			
0	18.0 (7/39)	1.00 (ref.)	1.00 (ref.)
1	15.8 (32/202)	0.88 (0.42–1.86)	0.84 (0.42–1.69)
2	15.8 (60/381)	0.88 (0.43–1.79)	0.81 (0.41–1.57)
3	16.9 (116/688)	0.94 (0.47–1.88)	0.86 (0.45–1.65)
≥4	11.9 (277/2330)	0.66 (0.34–1.31)	0.62 (0.33–1.18)
Per additional sibling	—	0.91 (0.87–0.95)	0.92 (0.88–0.96)
*P*_trend_	—	<0.001	<0.001

*Older siblings*			
0	14.4 (152/1055)	1.00 (ref.)	1.00 (ref.)
1	14.3 (112/784)	0.96 (0.77–1.21)	0.93 (0.74–1.16)
2	16.5 (101/614)	1.04 (0.82–1.32)	0.98 (0.77–1.23)
3	11.8 (57/485)	0.74 (0.55–0.99)	0.71 (0.53–0.95)
≥4	10.0 (74/743)	0.61 (0.45–0.81)	0.59 (0.44–0.80)
Per additional older sibling	—	0.90 (0.86–0.95)	0.90 (0.85–0.94)
*P*_trend_	—	<0.001	<0.001

*Younger siblings*			
0	13.1 (69/526)	1.00 (ref.)	1.00 (ref.)
1	13.9 (102/736)	0.98 (0.73–1.30)	0.97 (0.72–1.30)
2	14.9 (113/761)	0.98 (0.73–1.30)	1.02 (0.76–1.36)
3	14.8 (114/770)	0.92 (0.69–1.23)	0.94 (0.70–1.27)
≥4	11.1 (92/831)	0.68 (0.50–0.92)	0.74 (0.54–1.01)
Per additional younger sibling	—	0.93 (0.88–0.98)	0.94 (0.89–1.00)
*P*_trend_	—	0.008	0.050

PR: prevalence ratio; CI: confidence interval; ref.: reference. ^*∗*^PRs of older siblings were simultaneously adjusted for younger siblings, and PRs of younger siblings were simultaneously adjusted for older siblings. ^†^Adjusted for sex, age, body mass index, mode of birth, current cat exposure, dog exposure in infancy, environmental tobacco smoke exposure, parental history of rhinitis, parental history of asthma, and parental history of eczema. Additionally, PRs of older siblings were simultaneously adjusted for younger siblings, and PRs of younger siblings were simultaneously adjusted for older siblings.

## Data Availability

The data used to support the findings of this study are available from the corresponding author upon request.
